# Early type 1 diabetes aggravates renal ischemia/reperfusion-induced acute kidney injury

**DOI:** 10.1038/s41598-021-97839-7

**Published:** 2021-09-24

**Authors:** Mariana Charleaux de Ponte, Vanessa Gerolde Cardoso, Guilherme Lopes Gonçalves, Juliana Martins Costa-Pessoa, Maria Oliveira-Souza

**Affiliations:** Laboratory of Renal Physiology, Department of Physiology and Biophysics, Institute of Biomedical Sciences, University of Sao Paulo, São Paulo, SP 05508-900 Brazil

**Keywords:** Cell biology, Physiology, Nephrology

## Abstract

The present study aimed to investigate the interaction between early diabetes and renal IR-induced AKI and to clarify the mechanisms involved. C57BL/6J mice were assigned to the following groups: (1) sham-operated; (2) renal IR; (3) streptozotocin (STZ—55 mg/kg/day) and sham operation; and (4) STZ and renal IR. On the 12th day after treatments, the animals were subjected to bilateral IR for 30 min followed by reperfusion for 48 h, at which time the animals were euthanized. Renal function was assessed by plasma creatinine and urea levels, as well urinary protein contents. Kidney morphology and gene and protein expression were also evaluated. Compared to the sham group, renal IR increased plasma creatinine, urea and albuminuria levels and decreased *Nphs1* mRNA expression and nephrin and WT1 protein staining. Tubular injury was observed with increased Havcr*1* and Mki*67* mRNA expression accompanied by reduced megalin staining. Renal IR also resulted in increased SQSTM1 protein expression and increased proinflammatory and profibrotic factors mRNA expression. Although STZ treatment resulted in hyperglycemia, it did not induce significant changes in renal function. On the other hand, STZ treatment aggravated renal IR-induced AKI by exacerbating renal dysfunction, glomerular and tubular injury, inflammation, and profibrotic responses. Thus, early diabetes constitutes a relevant risk factor for renal IR-induced AKI.

## Introduction

Acute kidney injury (AKI) is a common disorder worldwide that is associated with high morbidity and mortality^1^. AKI due to renal ischemia and reperfusion (IR) occurs during various clinical conditions, such as cardiac and hepatic surgeries, shock, sepsis, vascular occlusions and kidney transplantation^2^. Under these circumstances, a rapid decline in kidney function occurs and is closely related to high plasma creatinine levels, renal epithelial and vascular cell injury, and interstitial innate immune cell infiltration; these phenomena lead to interstitial fibrosis and often to the development of chronic kidney disease (CKD), culminating in end-stage renal disease (ESRD)^3,4^. Moreover, the impairment of autophagy, a tubular protective mechanism, is associated with fibrosis extension and may facilitate the transition from AKI to CKD^5^. Despite recent advances in understanding the pathophysiological mechanisms associated with AKI, there is still no effective therapy for AKI prevention or treatment.

Diabetes mellitus is a metabolic syndrome generally characterized by elevated blood glucose levels. Type 1 diabetes mellitus (T1DM) occurs due to an autoimmune disease characterized by the selective destruction of β-pancreatic cells that synthesize and secrete insulin, a hormone responsible for the maintenance of glycemia under physiological conditions^6^. Diabetic kidney disease (DKD) is closely related to CKD progression and loss of kidney function^7^. DKD is initially characterized by hyperfiltration, microalbuminuria and inflammatory cell infiltration. In the irreversible state, podocyte injury and thickening of the glomerular basement membrane occurs, with a consequent decline in the glomerular filtration rate and macroalbuminuria^7^.

It is known that diabetes is an important risk factor for AKI^8^, especially in cardiac patients^9^ or patients with partial nephrectomy^10^, after renal infarction^11^ and in septic shock^12^. In addition, it has been shown that diabetic patients who suffer episodes of AKI are more likely to develop CKD^13^. Although the mechanisms by which diabetes influences the severity of renal IR injury remain unknown, some of the pathological findings in diabetic nephropathy, including interstitial inflammation and fibrosis, are also related to IR-induced AKI^14^.

Given the increased incidence of diabetes and its contribution as a risk factor for a variety of surgical complications^15,16^, more research is required to understand the molecular and cellular pathophysiological mechanisms underlying IR-induced AKI in diabetic conditions. In addition, studies with experimental models have associated the initial phase of ischemic AKI with chronic diabetes^17–19^, and little is known about the effects of AKI on the early stages of diabetes. Thus, the aim of the current study was to investigate the interaction between early diabetes and renal IR-associated AKI. Here, we established a renal IR model in streptozotocin (STZ)-induced diabetic mice to investigate whether hyperglycemia aggravates renal IR-induced AKI during early type 1 diabetes and the possible cellular mechanisms involved in this maladaptive process. In particular, we focused on kidney function, tubular and glomerular injury, autophagy, and gene expression related to inflammatory and fibrotic responses. In this study, we intend to contribute to the identification of possible therapeutic targets for the treatment of renal IR-induced AKI in early type 1 diabetic conditions.

## Results

### Initial body weight and blood glucose levels

Before surgery, all the animals were weighed, and the blood glucose levels were evaluated. STZ treatment did not change the body weight of the animals compared to the sham group [(g) sham 23.2 ± 1.2 (n = 16) vs STZ 22.4 ± 1.5 (n = 14), *p* = 0.5652]. However, STZ induced an increase in the blood glucose levels compared to the sham group [(mg/dL) sham 143.6 ± 19.2 (n = 16) vs STZ, 325.8 ± 39.7 (n = 14), p < 0.0001] This result confirms the effectiveness of the drug.

### Metabolic parameters after surgery

**Figure 1:**
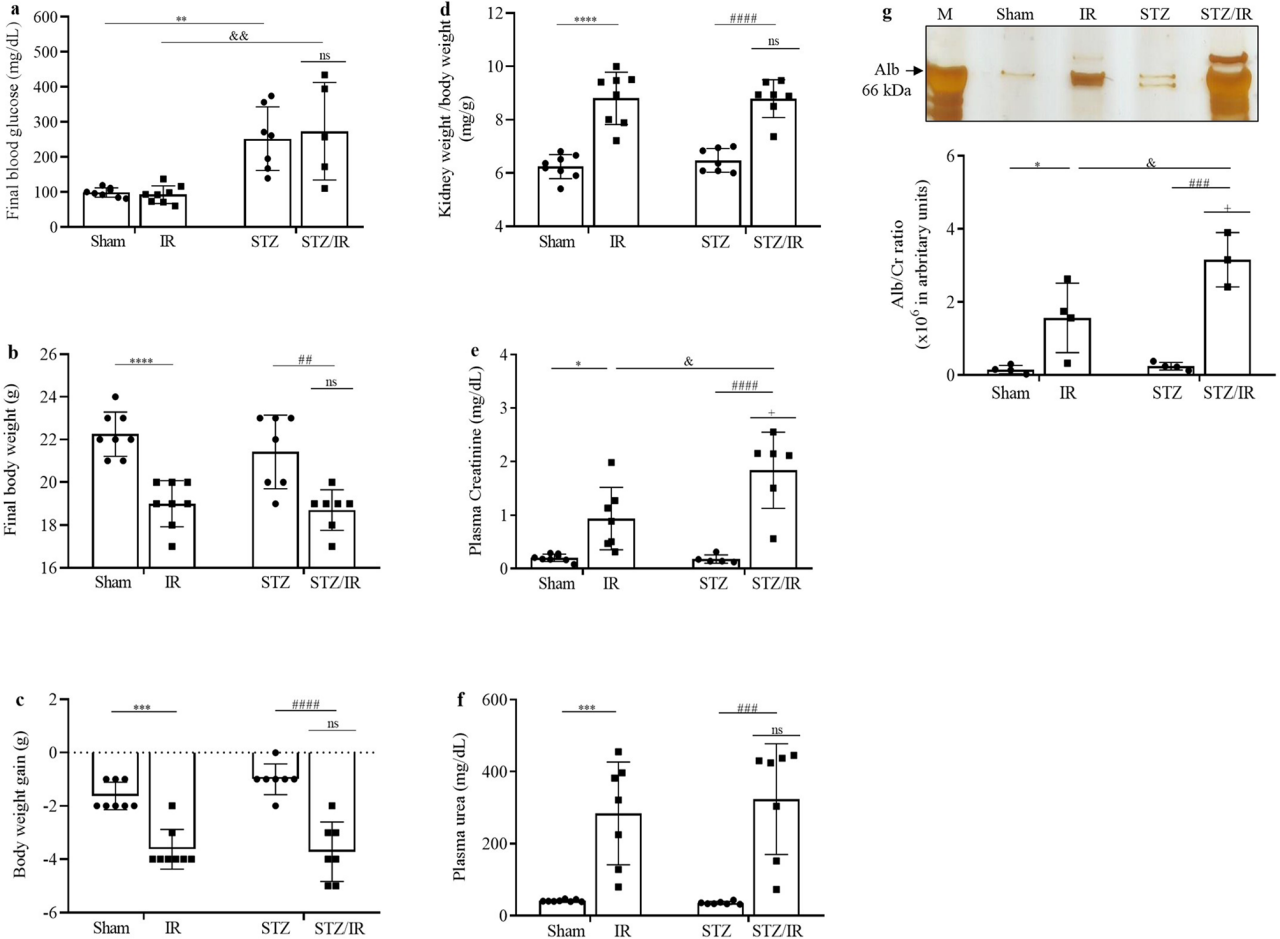
Effect of renal ischemia/reperfusion in vehicle- and STZ-treated mice on final blood glucose (**a**), final body weight (**b**), body weight gain (**c**), kidney weight/body weight (**d**), and plasma levels of creatinine (**e**), urea (**f**) and albuminuria (**g**). ^+^p < 0.05 for the interaction between diabetes and renal IR (STZ/IR), as indicated by two-way ANOVA (Supplementary Table S1). The Bonferroni post hoc test was also performed: *p < 0.05, **p < 0.01, ***p < 0.001, ****p < 0.0001 vs sham group; ^##^p < 0.01, ^###^p < 0.001, ^####^p < 0.0001 vs STZ group; ^&^p < 0.05, ^&&^p < 0.01 vs IR group. The values are the mean ± S.D. (n = 3–8). The samples were obtained from the same experiment. *Sham* sham-operated, *IR* ischemia/reperfusion, *STZ* streptozotocin, *STZ/IR* streptozotocin/ischemia/reperfusion, *Alb* albumin, *M* marker, *Cr* creatinine, *ns* nonsignificant.

To investigate the interference of early hyperglycemia in IR-induced AKI, we established a 12-day model of STZ-induced type 1 diabetes. As shown in Fig. 1a–d**,** multiple comparisons tests revealed that final blood glucose (FBG) levels were similar between the sham and IR groups, but as expected, in the STZ group, this parameter was significantly increased compared to that in the sham group and remained high in the STZ/IR group. In addition, a significant decrease in final body weight (FBW) and body weight gain (BWG) and an increase in kidney weight/body weight (KW/BW) were observed in the IR group compared to the sham group. STZ treatment did not change these parameters compared to the sham group and did not interfere with the IR response, as observed by two-way ANOVA, where the interactions were not significant (FBW, p = 0.5535; BWG, *p* = *0.2128*; KW/BW, *p* = 0.6252 and FBG, *p* = 0.6347). The average values are shown in Supplementary Table S1.

### Kidney function

Considering that acute renal IR and STZ treatment can induce changes in renal function, the creatinine and urea plasma levels, as well as the urinary albumin content, were evaluated. As shown in Fig. 1e–g and further illustrated in Supplementary Fig. S1, multiple comparisons tests revealed that renal IR, but not STZ treatment, resulted in increased plasma creatinine levels, urea levels, and albuminuria compared to the sham group. However, when the STZ-treated group was subjected to renal IR, the plasma creatinine levels and albuminuria, but not plasma urea levels, were robustly increased compared with those in the nondiabetic renal IR group. Notably, two-way ANOVA revealed a relevant interaction between hyperglycemia and renal IR on the creatinine plasma level and urinary albumin (creatinine, *p* = 0.018; urinary albumin, *p* = 0.034), but not on the plasma urea level (urea, *p* = 0.554). The average values are shown in Supplementary Table S1.

### Glomerular injury

Glomerular damage in early diabetes or IR-induced AKI is not commonly investigated. Indeed, in the current study, we did not observe a significant difference in glomerular area between the studied groups [(µm^2^) sham: 6932 ± 585 (n = 8); IR: 7012 ± 593 (n = 8); STZ: 7576 ± 616 (n = 7); STZ/IR: 7737 ± 744 (n = 7)] by multiple comparisons test or two-way ANOVA, where *p* = 0.863. Despite this result, we decided to analyze other glomerular parameters.

**Figure 2:**
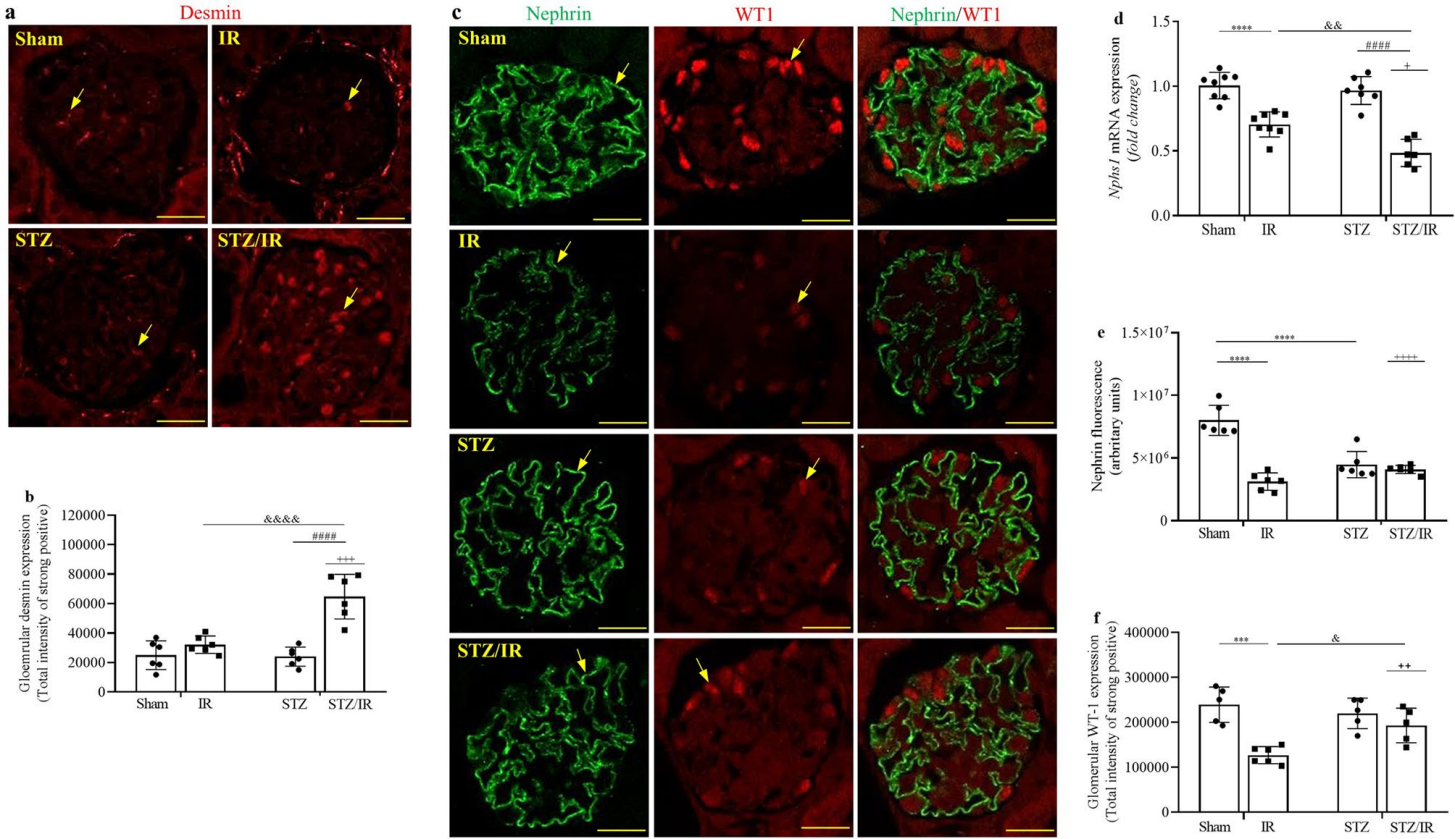
Effect of renal ischemia/reperfusion in vehicle- and STZ-treated mice on desmin staining (**a**,**b**), nephrin and WT1 staining (**c**) and Nphs1 (nephrin) mRNA expression (**d**). The averages of the fluorescence intensities for the nephrin and WT1 proteins are also represented (**e**,**f** respectively). ^+^p < 0.05, ^++^p < 0.01, ^+++^p < 0.001, ^++++^p < 0.0001 for the interaction between diabetes and renal IR (STZ/IR), as indicated by two-way ANOVA (Supplementary Table S2). The Bonferroni post hoc test was also performed: ***p < 0.001, ****p < 0.0001 vs sham group; ^####^p < 0.0001 vs STZ group; ^&^p < 0.05, ^&&^p < 0.01, ^&&&&^p < 0.0001 vs IR group. The values are the mean ± S.D. (n = 5–8). Immunofluorescence images were captured using a Zeiss LSM 510 confocal microscope equipped with a 63 × objective and laser excitation at 488 or 543 nm. The fluorescence intensity was analyzed using Aperio ImageScope software version 12.3.2. Bars = 50 µm. *Sham* sham-operated, *IR* ischemia/reperfusion, *STZ* streptozotocin, *STZ/IR* streptozotocin/ischemia/reperfusion.

As shown in Fig. 2a and Supplementary Fig. S2a, as well as Fig. 2b, multiple comparisons test analysis revealed that glomerular desmin staining was similar between the sham, IR and STZ groups. However, a significant interaction between STZ and IR in the increase of desmin staining was confirmed by two-way ANOVA (*p* = 0.001). Next, podocyte factors were also investigated, and as shown in Fig. 2c, Supplementary Fig. S2b and Fig. 2d–f, *Nphs1* (nephrin) mRNA expression was decreased in the IR group, while STZ treatment did not change this parameter compared to the sham group. In contrast, when STZ-treated mice were subjected to renal IR, a significant decrease in *Nphs1* mRNA expression was observed in comparison to the IR and STZ groups, and the interaction between STZ/IR was confirmed by two-way ANOVA (*p* = 0.0253). Next, nephrin protein staining was analyzed by multiple comparisons tests. The results revealed that compared to the sham group, both renal IR and STZ treatment resulted in a significant decrease in nephrin protein staining, combined with changes in protein organization throughout the glomerulus. In addition, a robust interaction between STZ treatment and renal IR was confirmed by two-way ANOVA (*p* < 0.0001). To confirm podocyte injury, we also investigated the glomerular WT1 distribution, and multiple comparisons tests revealed a significant decrease in WT1 staining in the IR group compared with the sham group. For example, as observed with nephrin expression, WT1 staining revealed a significant interaction between STZ treatment and renal IR, which was confirmed by two-way ANOVA (*p* = 0.0188). Merged nephrin and WT1 staining was also evident, and together, these results indicate that early hyperglycemia enhances podocyte injury in an IR-induced AKI model. The average values are shown in Supplementary Table S2.

### Tubular injury

**Figure 3:**
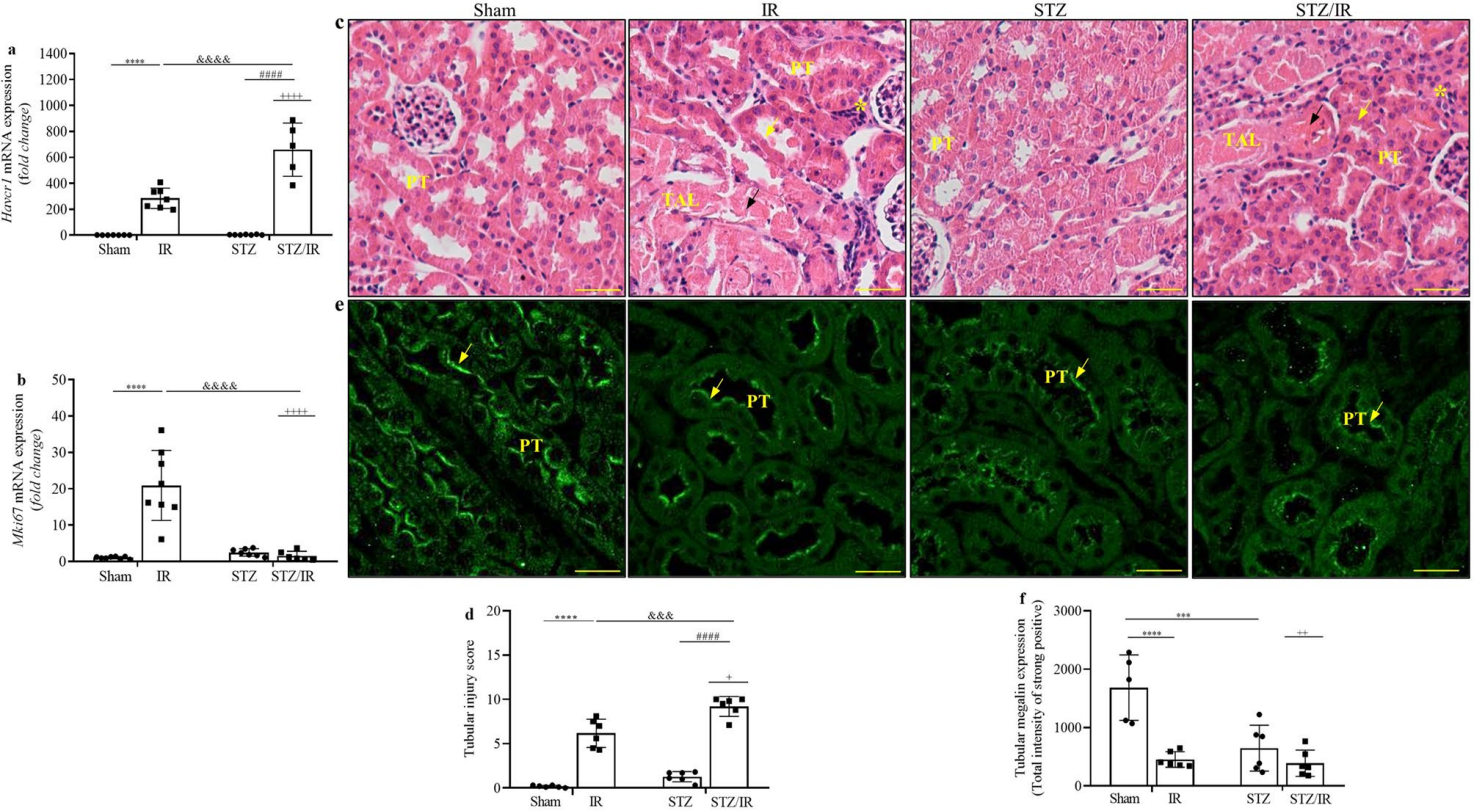
Effect of renal ischemia/reperfusion in vehicle- and STZ-treated mice on Havcr1 (Kim-1) (**a**) and Mki67 (Ki67) mRNA expression (**b**), as well as tubular morphology changes and megalin staining (**c**–**f**). ^+^p < 0.05, ^++^p < 0.01, ^++++^p < 0.0001 relative to the interaction between diabetes and renal IR (STZ/IR), as indicated by two-way ANOVA (Supplementary Table S2). The Bonferroni post hoc test was also performed: ***p < 0.001, ****p < 0.0001 vs sham group; ^####^p < 0.0001 vs STZ group; ^&&&^p < 0.001, ^&&&&^p < 0.0001 vs IR group. The black arrows indicate the formation of intratubular casts; the yellow arrows indicate loss of brush border and tubular epithelial cells; and the asterisks indicate interstitial infiltration. The values are the mean ± SD (n = 5–8). For morphological studies, fixed 4-µm-thick kidney slices were stained with hematoxylin–eosin, and the images were captured and analyzed using NIS-Elements (Nikon) software coupled to a light microscope equipped with a ×40 objective (Eclipse 80i, Nikon, Tokyo, Japan). Immunofluorescence images were captured using a Zeiss LSM 510 confocal microscope equipped with a ×40 objective and laser excitation at 488 or 543 nm. The fluorescence intensity was analyzed using Aperio ImageScope software version 12.3.2. Bars, 100 μm. *Sham* sham-operated, *IR* ischemia/reperfusion, *STZ* streptozotocin, *STZ/IR* streptozotocin/ischemia/reperfusion, *PT* proximal tubule, *TAL* thick ascending limb.

First, we investigated tubular injury markers, and as shown in Fig. 3a,b, multiple comparisons tests revealed that renal IR resulted in increased *Havcr*1 (Kim-1) and *Mki67* (Ki-67) mRNA expression, and STZ treatment did not change this parameter compared to the sham groups. However, in the STZ-treated group subjected to renal IR, *Havcr*1 mRNA expression was robustly increased, while for *Mki67* mRNA expression, this response was completely abolished when compared to the nondiabetic IR group. In addition, two-way ANOVA confirmed the interactions between STZ treatment and renal IR for both parameters (*Havcr1*, *p* = 0.0001, Mki67, *p* < 0.0001).

Next, 4-µm-thick hematoxylin and eosin-stained kidney slices were evaluated, and the results indicated that kidney morphology was similar between the sham and STZ-treated mice. However, in the IR and STZ/IR groups, there was prominent formation of intratubular casts (indicated by black arrows), loss of brush border and tubular epithelial cells (indicated by yellow arrows) and interstitial immune cell infiltration (indicated by asterisks) (Fig. 3c) and Supplementary Fig. S3a. Under the same conditions, the tubular injury score was determined, and multiple comparisons test analysis revealed a significant effect of renal IR on tubular injury, which was enhanced in the STZ-treated group subjected to renal IR, as confirmed by two-way ANOVA (*p* = 0.0311) (Fig. 3d).

We also investigated the tubular megalin contents (Fig. 3e and Supplementary Fig. S3b) and Fig. 3f. Using multiple comparisons tests, our results indicated a significant decrease in tubular megalin staining in the IR and STZ groups compared to the sham group. In addition, two-way ANOVA confirmed the interaction between STZ treatment and renal IR (*p* = 0.0038). The average values are shown in Supplementary Table S2.

### Autophagy

**Figure 4:**
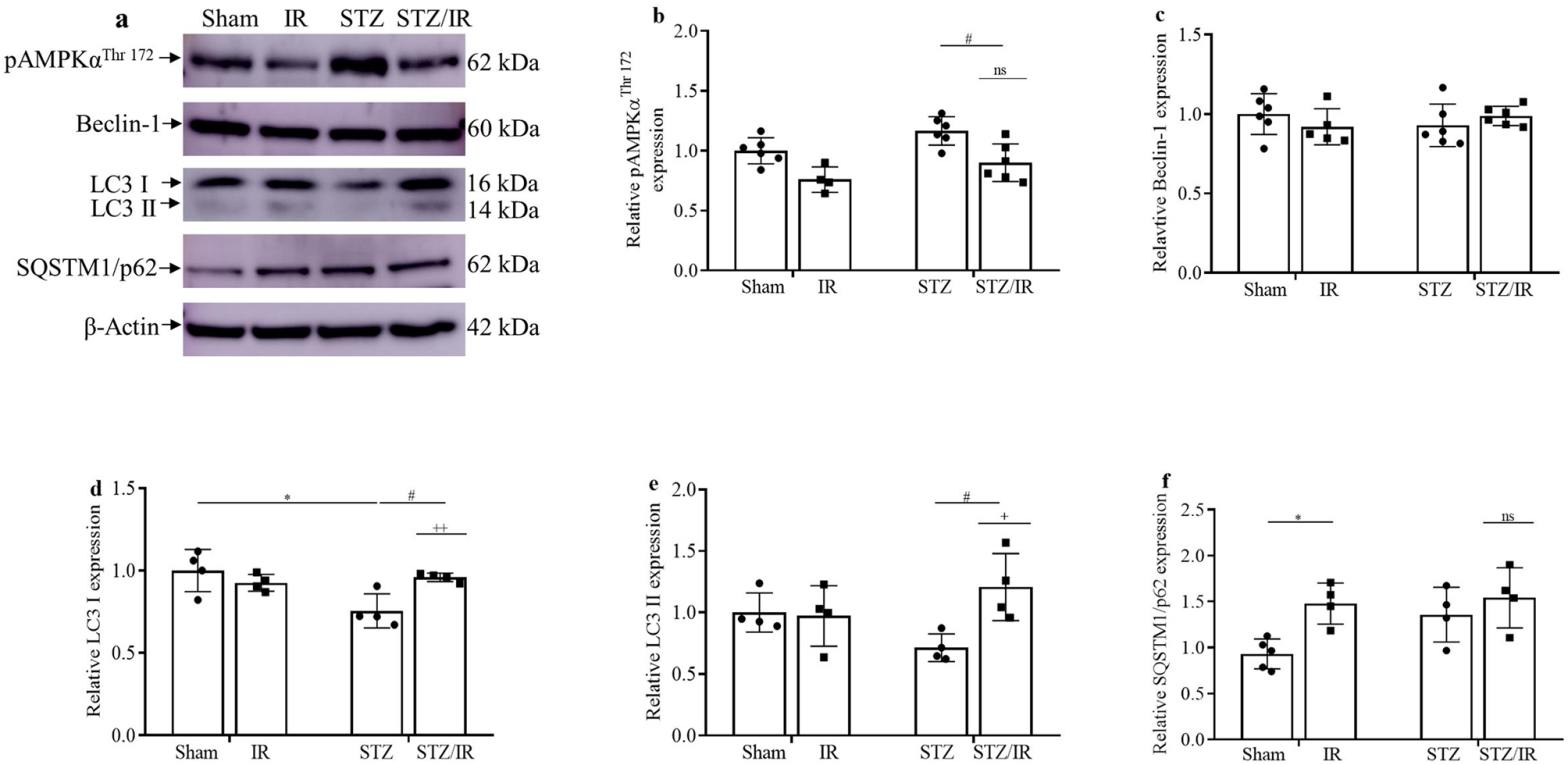
Effect of renal ischemia/reperfusion in vehicle- and STZ-treated mice on the protein expression (**a**) of phosphorylated AMPKα^Thr 172^ (**b**), beclin-1 (**c**), LC3 I (**d**), LC3 II (**e**), and SQSTM1/p62 (**f**). ^+^p < 0.05, ^++^p < 0.01 for the interaction between diabetes and renal IR (STZ/IR), as indicated by two-way ANOVA (Supplementary Table S2). The Bonferroni post hoc test was also performed: *p < 0.05 vs sham group; ^#^p < 0.05 vs STZ group. The values are the mean ± SD (n = 4–6). The samples were obtained from the same experiment, and the blots were processed in parallel. *Sham* sham-operated, *IR* ischemia/reperfusion, *STZ* streptozotocin, *STZ/IR* streptozotocin/ischemia/reperfusion, *ns* nonsignificant. β-actin: positive control.

Considering the renal injury observed and that autophagy is a conserved cellular recycling process, we evaluated the main autophagic components in our experimental models, focusing on AMPKα, beclin-1, LC3 I and II, and SQSTM1/p62 protein expression. As shown in Fig. 4a–f and further illustrated in Supplementary Fig. S4, our results obtained by multiple comparisons tests demonstrate that diabetic mice subjected to renal IR had decreased pAMPK^Thr172^ expression in comparison to the STZ group, but an interaction between STZ and IR was not observed by two-way ANOVA (pAMPK^Thr172^, p = 0.8201). In addition, total beclin-1 protein expression was similar among all the studied groups, and consequently, no interaction was observed by two-way ANOVA (Beclin-1, *p* = 0.1554). We also evaluated the expression of the LC3 proteins. By multiple comparisons tests, our results demonstrated that renal IR did not change the total protein expression of either LC3 I or LC3 II when compared to the sham group. In contrast, STZ treatment only induced a significant decrease in LC3 I but not LC3 II protein expression compared to the sham group. However, when renal IR occurred after STZ treatment, both LC3 I and LC3 II protein expression was similar to that observed in the sham group. An interaction between the STZ-treated group subjected to renal IR was confirmed by two-way ANOVA (LC3 I, *p* = 0.0077; LC3 II, *p* = 0.0275). In addition to these results, a significant effect of renal IR on increasing SQSTM1/p62 protein expression was observed by multiple comparisons test analysis. No interaction between the STZ-treated group subjected to renal IR was observed, as confirmed by two-way ANOVA (SQSTM1/p62, *p* = 0.1654). The average values are shown in Supplementary Table S2.

### Inflammation

**Figure 5:**
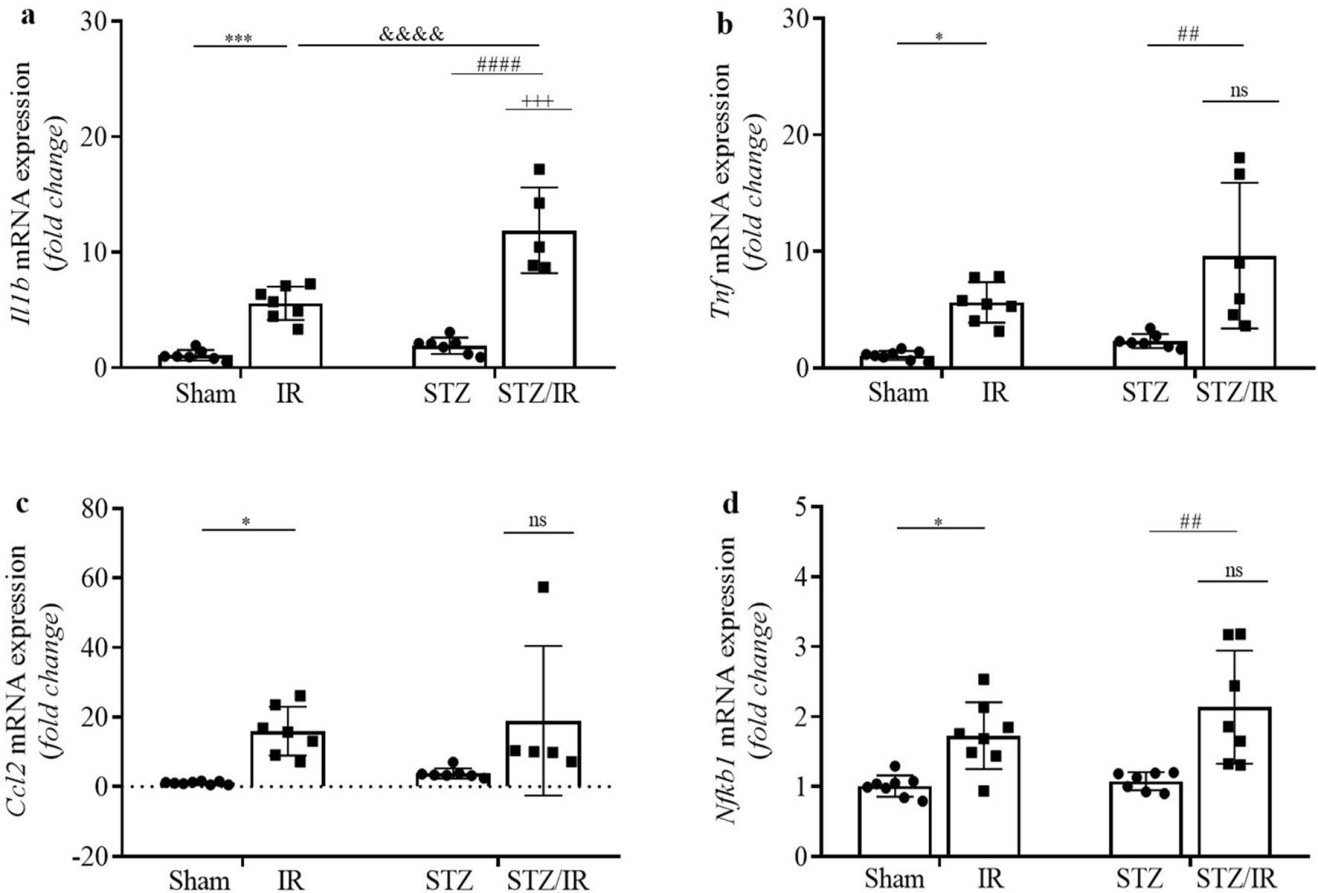
Effect of renal ischemia/reperfusion in vehicle- and STZ-treated mice on *Il1b* (IL-1β) (**a**), *Tnf (*TNF-α) (**b**), *Ccl2 (*MCP-1) (**c**), and *Nfkb1* (NFқB) (**d**) mRNA expression. ^+++^p < 0.001 for the interaction between diabetes and renal IR (STZ/IR), as indicated by two-way ANOVA (Supplementary Table S2). The Bonferroni post hoc test was also performed: *p < 0.05, ***p < 0.001 vs sham group; ^##^p < 0.01, ^####^p < 0.0001 vs STZ group; ^&&&&^p < 0.0001 vs IR group. The values are the mean ± SD (n = 5–8). *Sham* sham-operated, *IR* ischemia/reperfusion, *STZ* streptozotocin, *STZ/IR* streptozotocin/ischemia/reperfusion, *ns* nonsignificant.

Next, we investigated the gene expression of proinflammatory factors. As demonstrated in Fig. 5a–d, multiple comparisons tests indicated that renal IR resulted in a significant increase in *Il1b*, *Tnf*, *Ccl2* and *Nfkb1* mRNA expression compared to the sham group. STZ treatment did not alter the expression of these genes compared to the sham group. However, renal IR in the STZ-treated mice resulted in an amplification of *Il1b* but not *Tnf*, *Ccl2* or *Nfkb1* mRNA expression compared to that in the nondiabetic mice subjected to renal IR. Two-way ANOVA confirmed the interactions between the STZ-treated group subjected to renal IR only for *Il1b* mRNA expression (*Il1b*, *p* = 0.0009;* Tnfα*, *p* = 0.2379; *Ccl2*, *p* = 0.9720; *Nfkb1*, *p* = 0.3346). The average values are shown in Supplementary Table S2.

### Fibrosis

**Figure 6:**
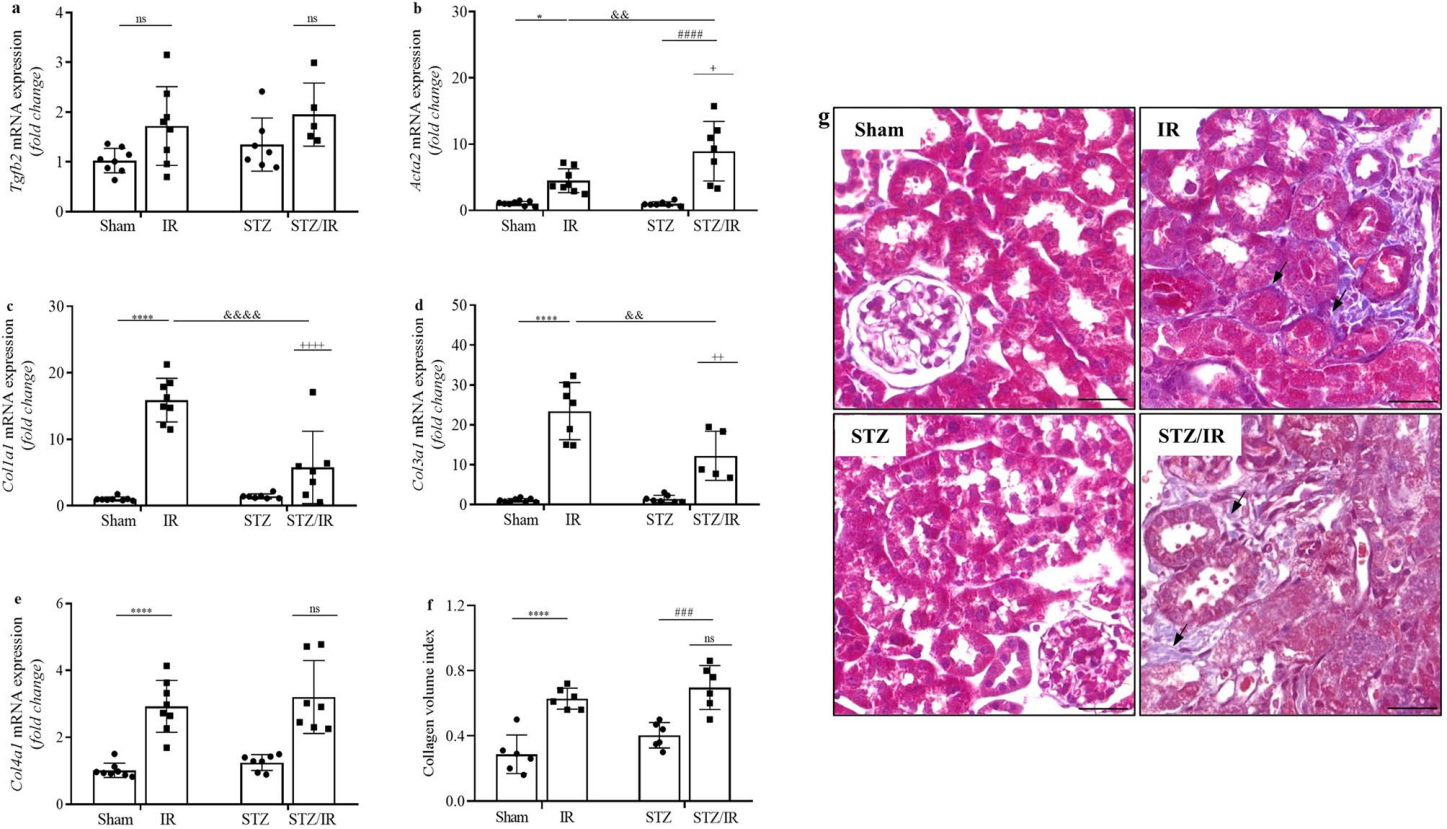
Effect of renal ischemia/reperfusion in vehicle- and STZ-treated mice on Tgfb2 (TGF-β2) (**a**), Acta2 (α-SMA) (**b**), Col1a1 (collagen I) (**c**), Col3a1 (collagen III) (**d**), and Col4a1 (collagen IV) (**e**) mRNA expression; interstitial collagen volume (**f**,**g**). ^+^p < 0.05, ^++^p < 0.01, ^++++^p < 0.0001 for the interaction between diabetes and renal IR (STZ/IR), as indicated by two-way ANOVA (Supplementary Table S2). The Bonferroni post hoc test was also performed: *p < 0.05, ****p < 0.0001 vs sham group; ^###^p < 0.001, ^####^p < 0001 vs STZ group; ^&&^p < 0.01, ^&&&&^p < 0.0001 vs IR group. The black arrows indicate interstitial collagen volume. The values are the mean ± SD (n = 5–8). For morphological studies, fixed 4-µm-thick kidney slices were stained with Masson’s trichrome, and the images were captured and analyzed using NIS-Elements (Nikon) software coupled to a light microscope equipped with a ×40 objective (Eclipse 80i, Nikon, Tokyo, Japan). Bars = 50 µm. *Sham* sham-operated, *IR* ischemia/reperfusion, *STZ* streptozotocin, *STZ/IR* streptozotocin/ischemia/reperfusion, *ns* nonsignificant.

Considering that inflammatory processes are associated with fibrosis, we investigated the mRNA expression of two profibrotic components: *Tgfb2* and *Acta2*. Multiple comparisons tests revealed that *Tgfb2* mRNA expression was similar between all studied groups (Fig. 6a). However, the renal IR group exhibited a significant increase in *Acta2* mRNA expression compared to the sham group. STZ treatment did not change this parameter compared to the sham group. However, renal IR amplified the increase in *Acta2* mRNA expression in the STZ-treated mice compared to that observed in the nondiabetic mice subjected to renal IR, as confirmed by two-way ANOVA (Fig. 6b) with *p* = *0.0150*. The average values are shown in Supplementary Table S2.

We also evaluated the mRNA expression of *Col1a1*, *Col3a1* and *Col4a1*. Using multiple comparisons tests, renal IR resulted in increased *Col1a1*, *Col3a1* and *Col4a1* mRNA expression compared to the sham group (Fig. 6c–e). STZ treatment did not change these parameters compared to the sham group. In contrast, when renal IR was combined with STZ treatment, the increase in the mRNA expression of both collagens *Col1a1* and *Col3a1* was less robust, while *Col4a1* did not change compared to the nondiabetic mice subjected to renal IR. The degree of interaction between the STZ-treated group subjected to renal IR for each parameter was confirmed by two-way ANOVA (*Col1a1*, *p* < 0.0001; *Col3a1*, *p* = 0.0032; *Col4a1*, *p* = 0.9224). In addition, Masson’s trichrome analysis confirmed these results, since the total collagen volume increased in the renal IR group. However, no interaction between the STZ-treated group subjected to renal IR was observed by two-way ANOVA (*Col1a1*, *p* = 0.5722) (Fig. 6f,g) and Supplementary Fig. S5. The average values are shown in Supplementary Table S2.

## Discussion

Studies have reported that DKD aggravates renal IR-induced AKI^18,20^. However, there is still a lack of studies that show an association of the early stages of diabetes with IR-induced AKI. By using STZ-induced diabetes and acute renal IR models, we observed that early diabetes may also aggravate IR-induced AKI by exacerbating glomerular and tubular injury, as well as proinflammatory and profibrotic mRNA expression. These conditions may be associated with a maladaptive repair process.

In our study, only renal IR-induced AKI resulted in a significant decrease in final body weight gain, probably due to difficulty eating food after surgery and increased kidney weight, indicating kidney tissue and/or interstitial injury. For the other groups, most of the metabolic parameters remained unchanged, except for the hyperglycemia observed in the diabetic groups, as expected.

We also observed a significant decline in kidney function in mice subjected to renal IR since, under this condition, the animals showed increased creatinine and urea plasma levels and albuminuria. Moreover, diabetes exacerbated the IR response on plasma creatinine levels and albuminuria compared to those in the nondiabetic renal IR group. The plasma creatinine level is an important marker of the glomerular filtration rate (GFR), although it can also be associated with other factors, such as the tubular secretion rate and urine volume^21,22^. Thus, in the current study, we also evaluated different factors related to glomerular injury. First, STZ treatment associated with renal IR resulted in increased glomerular desmin staining. In healthy glomeruli, desmin is distributed mainly in mesangial cells. However, in different experimental rodent models with glomerular disease, increased glomerular desmin staining is recognized as a marker of podocyte dedifferentiation and injury^23–25^. Then, glomerular injury in both the renal IR and STZ/IR groups was confirmed by a significant decrease in nephrin mRNA expression and nephrin and WT1 protein staining. Podocytes are terminally differentiated and nonproliferative glomerular epithelial cells that are situated on the outer surface of the glomerular capillary basement membrane (GBM)^26^. Under healthy conditions, podocyte foot processes are connected to each other by slit diaphragms (SDs), which are organized by different molecules, including nephrin^27–29^. In addition, WT1 is a transcription factor that regulates the differentiation state of podocytes and is highly expressed in mature podocytes^30^. It has been documented that injured podocytes re-enter the cell cycle, leading to dedifferentiation, podocyte damage and a loss of ultrafiltration barrier function, resulting in proteinuria^31^, which is the major risk factor for progressive kidney injury. Taken together, our results indicate that early diabetes aggravates the loss of kidney function and glomerular injury in renal IR-induced AKI.

Since albuminuria may be associated with both glomerular and tubular injury, we extended our observations to the renal tubules. Microscopic analysis revealed strong tubular cast formation in the groups subjected to renal IR. Tubular and urinary casts are classified as hyaline, granular, fatty, and leukocyte aggregates^32^ and appear in patients with acute tubular necrosis^33^. Relative to the proximal tubule (PT), the epithelium has a potent ability to proliferate and repair itself^34^, since proximal tubular epithelial cells (PTECs) express kidney injury molecule 1 (Kim-1), which is highly upregulated after acute kidney injury^35,36^ and acts as a marker of cell differentiation and proliferation^37^. In addition to Kim-1, Ki67 is used as a marker of tubular regeneration and renal repair after AKI^38,39^. Severe AKI could result in incomplete repair, and a persistent increase in Kim-1 and Ki67 expression in tubular cells leads to the AKI-to-CKD transition^40^.

In agreement with these findings, our results indicate that IR-induced AKI is associated with severe PTEC injury, since Kim-1 and Ki67 gene expression appeared to be significantly increased in the nondiabetic renal IR group, suggesting that after insult, there is a predisposition toward cell differentiation and proliferation, which are essential for subsequent tissue repair. Interestingly, in the STZ/IR group, Kim-1 gene expression was robustly increased, and in contrast, diabetes impaired Ki67 gene expression, suggesting that early diabetes combined with IR promotes a negative response to tubular cell proliferation, a condition that can lead to maladaptive tissue repair.

In the current study, proximal tubular injury was confirmed by decreased megalin protein expression in renal IR and diabetic animals, highlighting that diabetes exacerbates IR-induced megalin loss. Megalin is a large glycoprotein (~ 600 kDa) expressed mainly in PT cells that acts as an endocytic receptor involved in the reabsorption of various molecules, including albumin and other low-molecular-weight proteins from glomerular filtrates^41^. Together, these results indicate that diabetes may hinder renal tubular tissue repair after renal IR, contributing to albuminuria.

Next, we evaluated autophagy, since it is a dynamic process that is activated in response to various cellular stress conditions, including hypoxia, oxidative stress, nutrient deprivation and organelle damage^42^. Autophagic flux involves phagophore nucleation and elongation into an autophagosome, which engulfs intracellular damaged components and then fuses with the lysosome for subsequent hydrolysis^43^. Energy stress triggers autophagy in mammalian cells by activating the energy sensor AMP-activated protein kinase (AMPK), which induces autophagy by directly phosphorylating the uncoordinated-51-like protein kinase (ULK1/ATG1) complex at serine residues Ser317, Ser555, and Ser777^44,45^. Moreover, AMPK phosphorylates Beclin-1 at Ser91/94^46^ and mediates the inactivation of mammalian target of rapamycin (mTOR), a suppressor of autophagic flux^47^. The ULK1/AGT complex and beclin-1 initiate phagophore nucleation^42^. The elongation of the phagophore involves multiple protein complexes, including the ATG12 system and LC3 II, the latter of which is a protein specifically related to autophagosome formation. Simultaneously, cytosolic LC3 is cleaved to form LC3 I, which is conjugated to phosphatidylethanolamine (PE) by ATG7 and ATG3, facilitating the conversion of cytosolic LC3 I to membrane-bound LC3 II, which in turn maintains autophagosome formation^48^. The membrane protein LC3 II can interact with p62/SQSTM1, also referred to as sequestosome 1, which mediates the autophagic transport of ubiquitinated substrates, such as misfolded proteins, for degradation in autolysosomes^49^. Thus, p62 seems to be critical in the autophagic cascade since inhibition of autophagy is often accompanied by upregulation of p62 expression. In the healthy kidney, autophagy acts as a quality control system for cellular metabolism and organelle homeostasis. Under pathological conditions, such as renal IR-induced AKI, autophagy is activated but seems to be unable to provide protection from cellular stress^50^; however, the mechanisms are unknown. In this context, we evaluated the same proteins involved in autophagic flux in the injured kidneys of nondiabetic and diabetic animals with renal IR-induced AKI. Our results revealed that renal IR did not change phosphorylated AMPK, beclin-1 and LC3 I or LC3 II protein expression but increased p62 protein expression, which indicates a lack of an autophagic response. Moreover, diabetes induced a decrease in LC3 I, preventing LC3 II formation and consequently interfering with p62/SQSTM1 activity. Together, these results complement the above observations regarding Kim-1 and Ki67 and are consistent with the absence of autophagy during renal IR-induced AKI. Interestingly, early diabetes did not change the IR-induced effects on p62/SQSTM1 protein expression, which maintained the inability of kidney tissue to repair after renal IR insult.

It is known that the autophagy pathway may prevent tissue inflammation through its role in apoptotic cell clearance^51^. Thus, it is possible that the loss of autophagic machinery can potentiate the inflammatory response in many tissues. Consistent with these findings, we observed that renal IR resulted in increased gene expression of IL-1β, TNF-α, MCP-1, and NFκB compared to sham surgery, and only IL-1β gene expression was higher in the early diabetic group subjected to renal IR. Together, these results suggest the relevance of autophagy in preventing exacerbated inflammation in renal IR-induced AKI, highlighting the contribution of early diabetes to enhancing IL-1β gene expression compared to that observed in nondiabetic mice subjected to renal IR. Interestingly, the contribution of IL-1β has been reported not only in proximal tubular injury but also in fibrosis development^52^.

Considering the relationship between inflammation and fibrosis^53^, we next investigated the gene expression of profibrotic components such as *Tgfb2*, a key transcription factor associated with tubulointerstitial and glomerular fibrosis^54^, and *Acta2*, a commonly used marker of myofibroblasts^55^. Our results revealed that renal IR in both nondiabetic and diabetic mice resulted in increased *Acta2* gene expression compared to that in sham and STZ-treated mice, suggesting a fibrotic mRNA expression response. Furthermore, early diabetes amplified *Acta2* gene expression. These results are consistent with other studies that reported increased profibrotic mRNA expression during AKI^56^. Here, we highlight that early diabetes aggravated the profibrotic mRNA expression induced by renal IR. In contrast, α-SMA protein expression was similar between the studied groups (data not shown), suggesting that early diabetes combined with renal IR-induced AKI is a critical condition to begin myofibroblast recruitment, extracellular matrix deposition and subsequent tubulointerstitial fibrosis.

In accordance with these findings, our results revealed that in the renal IR group, collagen I and III gene expression was significantly increased. However, compared with nondiabetic mice subjected to renal IR, mice with early diabetes subjected to renal IR exhibited moderate *Col1a1* and *Col3a1* gene expression. In contrast, renal IR resulted in an increase in *Col4a1* gene expression, suggesting a compensatory mechanism of GBM against the possible loss of its components. Collagen IV is the main component of GBM, and its function is to provide structure and support for other cell types, such as podocytes^57^. In addition to profibrotic gene expression, we found increased collagen staining in ischemic animals, highlighting the contribution of these proteins to initiating the fibrotic process in early diabetes associated with IR-induced AKI.

In conclusion, our data suggest that early diabetes aggravates renal IR-induced AKI mainly by exacerbating glomerular and tubular injury, as well as proinflammatory and profibrotic responses. Our findings are relevant and can contribute to the understanding of the molecular mechanisms associated with renal IR-induced AKI in early diabetic conditions and may provide relevant information to prevent adverse outcomes in T1DM patients affected by renal IR.

## Methods

### Animals

Male C57BL/6J mice, acquired from the animal care facility of the University of Sao Paulo Medical School (Sao Paulo, Brazil), were housed at the facility of the Department of Physiology and Biophysics, Institute of Biomedical Sciences, University of Sao Paulo (Sao Paulo, Brazil). All animals were maintained under a 12:12-h light–dark cycle with free access to water and food. All the presented protocols were approved by the Ethics Committee on the Use of Experimental Animals (12/2017) and were conducted in accordance with the ethical principles adopted by the Brazilian Society of Laboratory Animal Science and in compliance with the ARRIVE guidelines.

Thirty mice (aged 8 weeks, weighing approximately 22 g) were randomly assigned to four groups: sham-operated (sham); renal IR (IR); streptozotocin (STZ); and STZ followed by renal IR (STZ/IR).

### Streptozotocin treatment

According to the Ethics Committee, fasting nonsedated animals received a daily intraperitoneal injection (55 mg/kg)^58^ of STZ (Sigma-Aldrich, St. Louis, MO, USA), diluted in citrate buffer (0.1 M, pH 4.5), for five consecutive days. Notably, the sham and IR groups received only citrate buffer (vehicle). On the fifth day after the last injection of STZ, asymptomatic mice with blood glucose ≥ 250 mg/dL (evaluated by the Accu-Chek Performa Kit, Sao Paulo, SP, Brazil) were considered diabetic and therefore allowed to continue the study.

### Renal ischemia surgery and reperfusion

On the twelfth day after the last injection of STZ, the animals were weighed, and the blood glucose levels were evaluated. Next, the animals were intraperitoneally anesthetized with ketamine (100 mg/kg) and xylazine (20 mg/kg, Virbac, Jurubatuba, SP, Brazil) and immediately placed on a hot plate to maintain a constant temperature at 37 °C. After total loss of pain reflexes, an abdominal incision was made in the white line to expose the kidneys. The blood flow of both renal arteries was interrupted by the use of mini-clamps (RS-5426, Roboz Surgical Instrument Company, Inc., Gaithersburg, MD, USA) for 30 min^59^. Renal ischemia was assessed based on the change in kidney color. Then, the mini-clamps were carefully removed to allow renal reperfusion, which was confirmed by the return of oxygenated blood to the kidneys. Subsequently, the abdominal muscles and skin were sutured, and asepsis was performed. The control and STZ groups underwent sham surgery, but the blood flow through the renal arteries was not interrupted. After surgery, the animals received saline solution intraperitoneally to prevent dehydration.

### Plasma, urine and kidney collection

At the end of 48 h of kidney reperfusion, the animals were weighed, and blood glucose was evaluated. The animals were then anesthetized as previously described, cardiac puncture was performed to collect blood samples (approximately 1 mL), and the mice were euthanized by exsanguination. Immediately, an abdominal incision was made using a scalpel, and the urinary content of the bladder was collected. Next, the left kidney was removed, weighed, and cut into two sections. One section was quickly frozen and pulverized in liquid nitrogen for further analysis by qPCR, and the remaining section was crushed in PBS solution (0.15 M NaCl containing 10 mM sodium phosphate buffer, pH 7.4) with protease inhibitors (Roche Laboratories, Basel, Switzerland), centrifuged (4000×*g* for 10 min at 4 °C), and the supernatant was frozen at − 80 °C for protein analysis by western blotting. The right kidney was perfused with PBS solution, removed, cross-sectioned, inserted into properly labeled histological cassettes and fixed in 4% paraformaldehyde solution. After dehydration, the slices were embedded in paraffin for morphological studies as previously described^60^.

### Kidney function analysis

The plasma creatinine and urea levels as well as urine creatinine levels were assessed using colorimetric tests (Labtest, Lagoa Santa, MG, Brazil) according to the manufacturer’s instructions. Urine osmolarity was measured using an osmometer (Micro osmometer, Precision Systems, Natick, MA, USA). The urinary albumin concentration was determined using a SilverQuest Silver Staining Kit (Sigma-Aldrich) according to the modified Oakley method^61^. Briefly, urine samples from the metabolic cages were separated by SDS polyacrylamide gel electrophoresis (10%). Next, silver staining was performed on the gels, and the albumin (bovine serum albumin—BSA; 66 kDa) bands were identified using a molecular weight marker. The experiments were carried out following the manufacturer’s instructions, and the urine protein concentration was normalized to the urine creatinine concentration. The bands were assessed by optical densitometry using ImageJ software (National Institutes of Health (NIH), Bethesda, MD, USA)^56^.

### Morphological studies

As previously described^56,60^ and summarized here, fixed 4-µm-thick kidney slices were deparaffinized and stained with hematoxylin–eosin, and histological studies were performed using NIS-Elements (Nikon) software coupled to a light microscope (Eclipse 80i, Nikon, Tokyo, Japan). For glomerular area analyses, 20 glomeruli per animal were manually surrounded, and the images provided the area values. Under the same conditions, cortical injured tubules were counted in 10 fields per animal using the following criteria: dilation, cell loss and formation of casts compatible with necrosis. Interstitial injury was assessed using kidney sections stained with Masson’s trichrome. For this assessment, approximately 10 fields per animal were graded as follows: level 0, without fibrosis; level 1, interstitial fibrotic area of 1–25%; level 2, interstitial fibrotic area of 26–50%; level 3, interstitial fibrotic area of 51–75%; and level 4, interstitial fibrotic area > 75%. The interstitial fibrotic area (IFA) index was calculated using the following formula: IFA = [(1 × NA1) + (2 × NA2) + (3 × NA3) + (4 × NA4)]/(NA0 + NA1 + NA2 + NA3 + NA4), where NA corresponds to the number of areas in each level. All the parameters above were analyzed in a blinded manner by different investigators.

### Total renal tissue gene expression studies

As previously described^23,60^ and summarized here, frozen kidney sections were homogenized in liquid nitrogen and then resuspended in TRIzol LS Reagent (Invitrogen, Carlsbad, CA USA) for RNA extraction (GE Healthcare Life Sciences, BW, Germany) according to the manufacturer’s instructions. Two hundred nanograms of RNA was used for the synthesis of cDNA (RT-PCR) with the High-Capacity RNA-to-cDNA kit (Applied Biosystems, Foster City, CA, USA) and Veriri thermocycler (Applied Biosystems), according to the product protocol. The cDNA (25 ng) obtained was used for the analysis of gene expression through real-time polymerase chain reaction (PCR) (StepOnePlus Real Time PCR System, Applied Biosystems). The following FAM-labeled probes obtained in the TaqMan Gene Expression Assays format (Applied Biosystems) were used: nephrin (*Nphs1*), Mm01176615_g1; kidney injury molecule-1, Kim-1 (*Havcr1*), Mm00506686_m1; antigen Ki67 (*Mki67*), Mm01278617_m1; interleukin 1 beta (*Il1b*), Mm00434228_m1; tumor necrosis factor alpha (*Tnf*α), Mm00443258_m1; monocyte chemoattractant protein-1, MCP1 (*Ccl2*), Mm00441242_m1; nuclear factor kappa B 1 (*Nfkb1*), Mm00476361_m1; alpha smooth muscle actin, αSMA (*Acta2*), Mm00725412_s1; transforming growth factor beta 2 (*Tgfb2*), Mm00436955_m1; collagen type I (*Col1a1*), Mm00801666_g1; collagen type III (*Col3a1*), Mm01254476_m1; collagen type IV (*Col4a1*), Mm01210125_m1; and glyceraldehyde-3-phosphate dehydrogenase (*Gapdh*), Mm99999915_g1 (reference gene). Real-time PCRs were performed in duplicate, and the data were analyzed by the comparative cycle threshold (2^ΔΔCt^) method. The results were normalized to *Gapdh* expression and are shown as the fold change relative to the sham group.

### Western blotting analysis

Immunoblotting analysis was performed on 30-μg protein aliquots resolved by 10–15% SDS-PAGE. Then, the protein samples were transferred to polyvinylidene fluoride (PVDF) membranes (GE Healthcare Life Sciences). The membranes were incubated with primary antibodies against autophagic markers such as rabbit monoclonal anti-AMPKα (1:1000, Cell Signaling, Danvers, MA, USA^62^), rabbit monoclonal anti-phospho-AMPKα^Thr172^ (1:1000, Cell Signaling^63^), monoclonal rabbit anti-Beclin-1 (1:1000, Cell Signaling^64^), rabbit monoclonal anti-LC3A/B (1:1000, Cell Signaling^65^), rabbit monoclonal anti-SQSTM1/p62 (1:1000, Cell Signaling^66^) and rabbit monoclonal anti-β-actin (1:8000, Abcam). Then, the membranes were incubated with conjugated goat secondary antibodies against rabbit or mouse (Jackson ImmunoResearch Laboratories, Baltimore, MD, USA). After that, the membranes were washed and exposed to the reagent for immunodetection with ECL™ luminescence (Amersham, GE) and subsequently to a photodocumenter (Amersham Imager 600, GE). The intensity of the bands was assessed by optical densitometry using ImageJ software (National Institute of Health). Protein expression was quantified relative to the expression of the endogenous control β-actin, and the results are presented as protein expression relative to the sham group.

### Immunofluorescence

The immunofluorescence experimental protocol was previously described^56^ and is summarized here. Fixed 4-µm-thick kidney sections were deparaffinized and blocked with 1% BSA and 10% goat serum in TBS (for rabbit and mouse primary antibodies) or just 1% BSA in TBS (for goat primary antibodies) for 2 h at room temperature. Next, the sections were incubated overnight at 4 °C with primary antibodies [goat anti-nephrin (1:100, R&D Systems, Minneapolis, MN, USA), rabbit anti-WT1 (1:200, Thermo Fisher Scientific, Waltham, Massachusetts, USA), rabbit anti-desmin (1:100, Abcam, Cambridge, MA, UK), and goat anti-megalin (1:50, Santa Cruz Biotechnology, Dallas, TX, USA)] and then with conjugated secondary antibodies. After that, the kidney sections were incubated with Alexa Fluor 594-conjugated F(ab′) goat anti-rabbit (1:200) and Alexa Fluor 488-conjugated F(ab′) rabbit anti-goat (1:200) secondary antibodies (Thermo Fisher, Rockford, USA) for 1 h at room temperature in the dark and mounted with Fluoroshield (Sigma-Aldrich). Protein staining was analyzed using a Zeiss LSM 510 confocal microscope equipped with a 40 × or 63 × objective and laser excitation at 488 or 543 nm. For nephrin, the positive glomeruli were evaluated in 10 fields per kidney, and the fluorescence intensity was analyzed using ImageJ software (National Institutes of Health—NIH, Bethesda, MD, USA). For WT1, desmin and megalin, the fluorescence intensity was analyzed using Aperio ImageScope software version 12.3.2 (Leica Biosystems, Buffalo Grove, IL, United States), and protein staining is expressed as the total intensity of the strong positive signal. All immunofluorescence analyses were performed blindly by one independent investigator, and the antibodies were validated by the manufacturer’s information through datasheets.

### Statistical analysis

The results presented as the mean ± S.D. were analyzed by two-way analysis of variance (ANOVA) using GraphPad Prism 8.0 software (GraphPad Software, Inc., San Diego, CA, USA). The interaction between two factors [ischemia reperfusion (IR) and streptozotocin (STZ)] was evaluated, and a Bonferroni post hoc test was performed for multiple comparisons. When analysis between two groups was necessary, a t test was performed. p < 0.05 was considered to indicate a statistically significant difference.

### Statement of ethics

All the experimental protocols were conducted in accordance with the ethical standards approved by the Institutional Animal Care and Use Committee of the University of Sao Paulo (Protocol no. 12/2017).

## Supplementary Information

Supplementary Information.

## Data Availability

All data generated or analyzed during this study are included in this published article (and its Supplementary Information Files).
