# Supplementary material for: Early type 1 diabetes aggravates renal ischemia/reperfusion-induced acute kidney injury
Source: Sci Rep. 2021 Sep 24;11:19028. doi: 10.1038/s41598-021-97839-7 (PMC8463569; doi:10.1038/s41598-021-97839-7)
Supplement: Supplementary file 1 — Supplementary Information. [file 41598_2021_97839_MOESM1_ESM.pdf]

Supplementary Material

Early type 1 diabetes aggravates renal ischemia/reperfusion-induced acute kidney injury

Mariana Charleaux de Ponte, Vanessa Gerolde Cardoso, Guilherme Lopes Gonçalves, Juliana Martins Costa-Pessoa and Maria Oliveira-Souza*

Laboratory of Renal Physiology, Department of Physiology and Biophysics, Institute of Biomedical Sciences, University of Sao Paulo, Sao Paulo, Brazil

***Corresponding author**

Prof. Maria Oliveira-Souza, Ph.D.

Laboratory of Renal Physiology, Department of Physiology and Biophysics, Institute of Biomedical Sciences, University of Sao Paulo, SP 05508-900, Brazil

Telephone number: 55 (11) 3091-8043

Fax number: 55 (11) 3091-7285

E-mail: souza@icb.usp.br

Supplementary Table S1: *Physiological parameters.*

Parameters	Multiple Comparisons				Two-way ANOVA	
	**Sham (8)**	**IR (8)**	**STZ (7)**	**STZ/IR (5-7)**	**STZ/IR**	***p-value***
Final blood glucose, mg/dL	98.5 ± 13.0	92.5 ± 25.3	251.8 ± 90.7**	273.4 ± 139.2$^{\&\&}$	F (1,24) = 0.2316^{ns}	*p = 0.6347*
Final body weight, g	22.2 ± 1.0	19.0 ± 1.0****	21.4 ± 1.7	18.7 ± 0.9$^{\#\#}$	F (1, 26) = 0.3604^{ns}	*p = 0.5535*
Body weight gain, g	-1.6 ± 0.5	-3.6 ± 0.7***	-1.0 ± 0.5	-3.7 ± 1.1$^{\#\#\#\#}$	F (1, 26) = 1.631^{ns}	*p = 0.2128*
Kidney weight /body weight, mg/g	6.24 ± 0.45	8.80 ± 0.98****	6.47 ± 0.44	8.78 ± 0.71$^{\#\#\#\#}$	F (1,26) = 0.2444^{ns}	*p = 0.6252*
**Parameters**	**Sham (4-8)**	**IR (4-7)**	**STZ (4-7)**	**STZ/IR (3-6)**	**STZ/IR**	***p-value***
Plasma Creatinine, mg/dL	0.203 ± 0.068	0.939 ± 0.582*	0.179 ± 0.078	1.839 ± 0.709$^{\#\#\#\#\&}$	F (1,22) = 6.432^{+}	*p = 0.018*
Plasma Urea, mg/dL	41.49 ± 2.82	283.95 ± 142.90***	35.29 ± 4.01	323.61± 153.91$^{\#\#\#}$	F (1,25) = 0.358 ^{ns}	*p = 0.554*
Urinary albumin, Alb/Cr ratio	0.155 ± 0.114	1.569 ± 0.951*	0.247 ± 0.106	3.159 ± 0.742$^{\#\#\#\&}$	F (1,11) = 5,871^{+}	*p = 0.034*

Two-way ANOVA with the Bonferroni multiple comparisons test was performed to compare data (means ± SDs). The number of animals per group is indicated in parentheses. For multiple comparisons: *p<0.05, ***p<0.001, ****p<0.0001 *vs* sham; $^{\#\#}$p<0.01, $^{\#\#\#}$p<0.001, $^{\#\#\#\#}$p<0.0001 *vs* STZ group; $^{\&}$p<0.05, $^{\&\&}$p<0.01 *vs* IR group. The F values and significance of the two-way ANOVA considering the interaction between renal ischemia/reperfusion (IR) and STZ treatment are given: $^{+}$p<0.05. Ns: nonsignificant; p: p value; g: grams.

Supplementary Table S2: *Genes and proteins expression.*

Parameters	Multiple Comparisons					Two-way ANOVA
**mRNA expression (fold change)**	**Sham (7-8)**	**IR (7-8)**	**STZ (7)**	**STZ/IR (5-7)**	**STZ/IR**	***p-value***
Nphs1	1.00 ± 0.10	0.70 ± 0.09****	0.96 ± 0.10	0.48 ± 0.10$^{\#\#\#\#\&\&}$	F (1,25) = 5.656^{+}	*p = 0.0253*
Havcr1	1.09 ± 0.53	284.70 ± 78.70****	3.90 ± 2.54	659.18 ± 205.13$^{\#\#\#\#\&\&\&\&}$	F (1,22) = 23.520^{++++}	*p<0.0001*
Mki67	1.04 ± 0.31	20.91 ± 9.65****	2.49 ± 1.00	1.55 ± 1.27$^{\&\&\&\&}$	F (1,25) = 29.000^{++++}	*p<0.0001*
Il1b	1.08 ± 0.46	5.58 ± 1.44***	1.90 ± 0.71	11.89 ± 3.71$^{\#\#\#\#\&\&\&\&}$	F (1,22) = 14.610^{+++}	*p = 0.0009*
Tnf	1.06 ± 0.38	5.62 ± 1.74*	2.30 ± 0.60	9.63 ± 6.25$^{\#\#}$	F (1,24) = 1.465^{ns}	*p = 0.2379*
Ccl2	1.08 ± 0.42	15.94 ± 7.00*	3.84 ± 1.47	18.97 ± 21.48	F (1,23) = 0.001^{ns}	*p = 0.9720*
Nfkb1	1.01 ±0.15	1.72 ± 0.47*	1.07 ± 0.13	2.13 ± 0.80$^{\#\#}$	F (1,26) = 0.966^{ns}	*p = 0.3346*
Tgfb2	1.02 ± 0.24	1.72 ± 0.79	1.34 ± 0.53	1.95 ± 0.63	F (1,24) = 0.043^{ns}	*p = 0.8376*
Acta2	1.04 ± 0.32	4.48 ± 1.79*	0.99 ± 0.32	8.94 ± 4.50$^{\#\#\#\#\&\&}$	F (1,24) = 0.043^{ns}	*p = 0.0150*
Col1a1	1.03 ± 0.31	15.90 ± 3.28****	1.43 ± 0.36	5.77 ± 5.44$^{\&\&\&\&}$	F (1,26) = 6.783^{+}	*p<0.0001*
Col3a1	1.07 ± 0.45	23.43 ± 7.16****	1.43 ± 0.91	12.22 ± 6.16$^{\#\#\&\&}$	F (1,23) = 10.820^{++}	*p = 0.0032*
Col4a1	1.01 ± 0.21	2.92 ± 0.77****	1.24 ± 0.23	3.20 ± 1.09$^{\#\#\#\#}$	F (1,26) = 0.009^{ns}	*p = 0.9224*
**Protein Staining**	**Sham (5 - 6)**	**IR (6)**	**STZ (5-6)**	**STZ/IR (5-6)**	**STZ/IR**	***p-value***
Desmin, IF	25020 ± 9756	32109 ± 5900	24033 ± 6439	64750 ± 15234$^{\#\#\#\#\&\&\&\&}$	F (1,20) = 16.810^{+++}	*p=0.0006*
Nephrin, IF	8007761±118401	3119389±694342****	4478745±105193****	4091277 ± 327249	F (1,20) = 39.240^{++++}	*p<0.0001*
WT1, IF	239201±39410	126686±19018***	219889 ±33969	192971 ±38535$^{\&}$	F (1,17) = 8.742^{++}	*p = 0.0088*
Megalin, IF	1684 ±561	453 ± 131****	647 ± 393***	392 ± 225	F (1,19) = 10.880^{++}	*p = 0.0038*
Tubular injury score	0.167 ±0.121	6.167 ± 1.600****	1.267 ± 0.582	9.200 ± 1.122$^{\#\#\#\#\&\&\&}$	F (1,20) = 5.376^{+}	*p = 0.0311*
Collagen index	0.287 ± 0.119	0.628 ± 0.065****	0.403 ± 0.078	0.697 ± 0.135$^{\#\#\#}$	F (1,20) = 0.3298^{ns}	*p = 0.5722*
**Protein/β-actin ratio**	**Sham (4-6)**	**IR (4-5)**	**STZ (4-6)**	**STZ/IR (4-6)**	**STZ/IR**	***p-value***
AMPKα	1.00 ± 0.12	0.91 ± 0.25	0.91 ± 0.17	0.92 ± 0.21	F (1,15) = 0,2857^{ns}	*p = 0.6008*
pAMPKα^{Thr172}	1.00 ± 0.10	0.76 ± 0.10*	1.16 ± 0.11	0.90 ± 0.15$^{\#}$	F (1,18) = 0.053^{ns}	*p = 0.8201*
Beclin-1	1.00 ± 0.12	0.92 ± 0.11	0.92 ± 0.13	0.98 ± 0.06	F (1,19) = 2.189^{ns}	*p = 0.1554*
LC3 I	1.00 ± 0.12	0.92 ± 0.05	0.75 ± 0.10*	0.95 ± 0.02$^{\#}$	F (1,12) = 10.230^{++}	*p = 0.0077*
LC3 II	1.00 ± 0.16	0.97 ± 0.24	0.71 ± 0.11	1.20 ± 0.27$^{\#}$	F (1,12) = 6.293^{+}	*p = 0.0275*
SQSTM1/p62	0.93 ± 0.16	1.47 ± 0.22*	1.35 ± 0.29	1.54 ± 0.32	F (1,13) = 2.160^{ns}	*p = 0.1654*

Two-way ANOVA with the Bonferroni multiple comparisons test was performed to compare data (means ± SDs). The number of animals per group is indicated in parentheses. For multiple comparisons: *p<0.05, ***p<0.001 ****p<0.0001 *vs* sham; $^{\#}$p<0.05, $^{\#\#}$p<0.01, $^{\#\#\#}$p<0.001, $^{\#\#\#\#}$p<0.0001 *vs* STZ group; $^{\&}$p<0.05, $^{\&\&}$p<0.01, $^{\&\&\&}$p<0.001, $^{\&\&\&\&}$p<0.0001 *vs* renal ischemia (IR) group. The F values and significance of the two-way ANOVA considering the interaction between renal ischemia/reperfusion (IR) and STZ treatment are given: $^{+}$p<0.05, $^{++}$p<0.01, $^{+++}$p<0.001, $^{++++}$p<0.0001. *Nphs1*: nephrin; *Havcr* 1: Kim-1; *Mki67*: Ki-67; *Il1b*: IL-1β; *Tnf*: TNF-α; *Ccl2*: MCP-1; *Nfkb1*: NFκB1; *Tgfb2*: TGF-β2; *Acta2*: α-SMA; *Col1a1*: Collagen I; *Col3a1*: Collagen III; *Col4a1*: Collagen IV; IF: immunofluorescence; ns: nonsignificant.

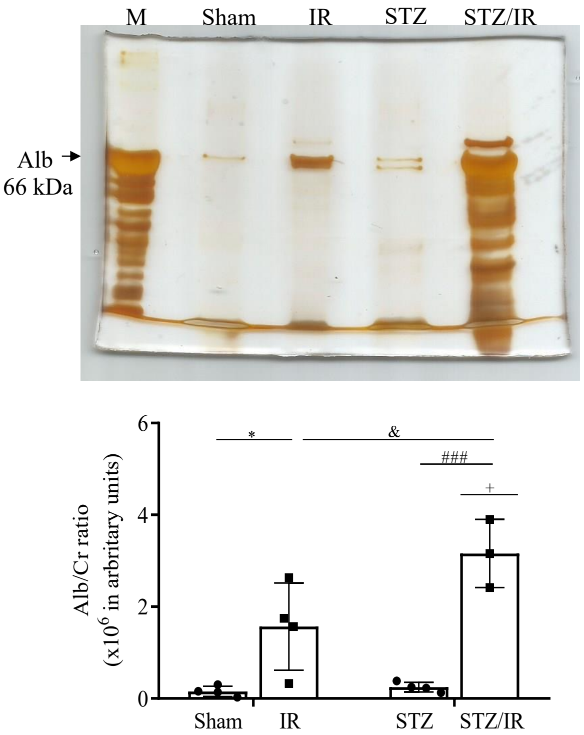

<u>Supplementary Fig. S1</u>: Effect of renal ischemia/reperfusion in vehicle- and STZ-treated mice on albuminuria. Representative full-length gel as well as the averages of albumin bands intensities. $^{+}p<0.05$ for the interaction between diabetes and renal IR (STZ/IR), as indicated by two-way ANOVA (Supplementary Table **S1**). The Bonferroni post hoc test was also performed: $^{*}p<0.05$ *vs* sham group; $^{###}p<0.001$ *vs* STZ group; $^{\&}p<0.05$ vs IR group. The values are the mean ± S.D. (n = 3-8). Sham: sham-operated; IR, ischemia reperfusion; STZ: streptozotocin; STZ/IR: streptozotocin/ischemia/reperfusion; Alb: albumin; M: marker.

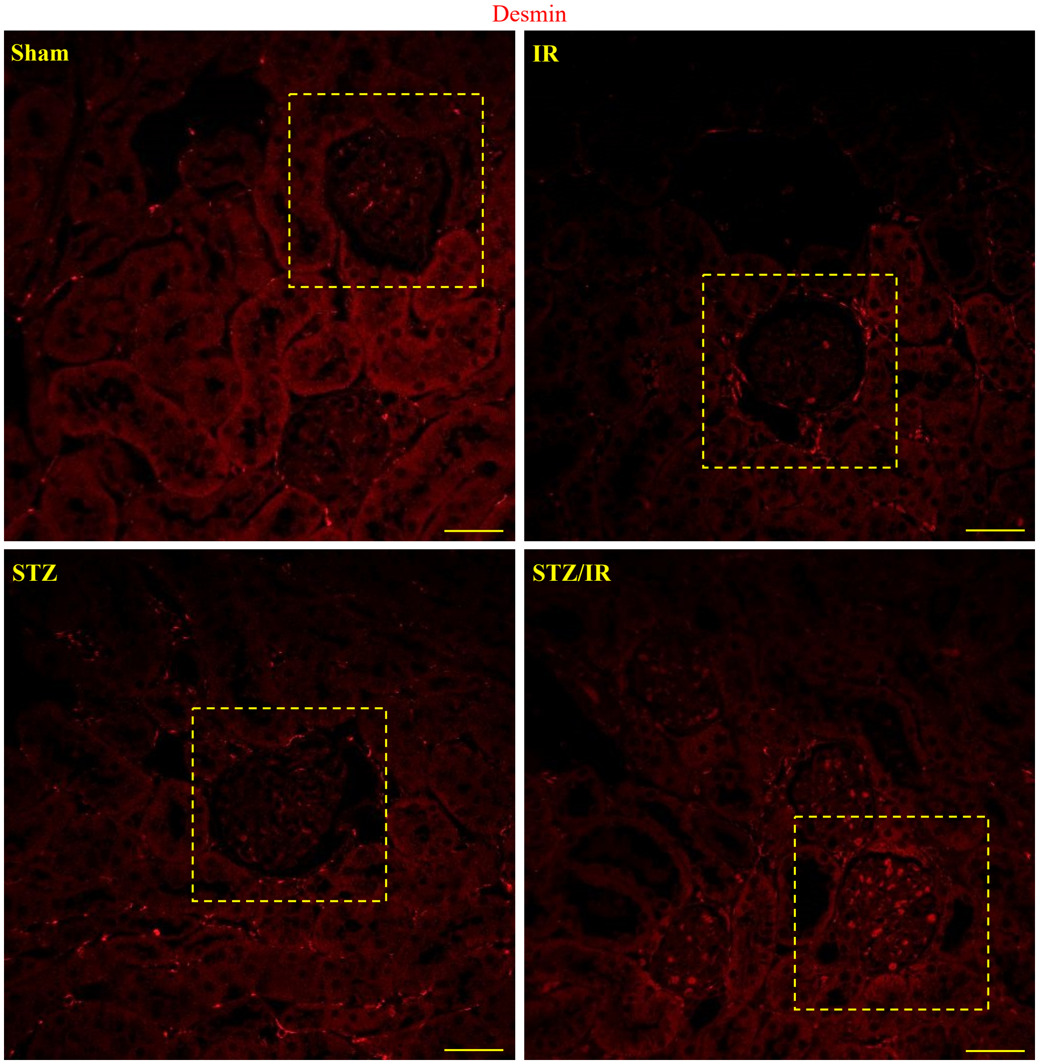

<u>Supplementary Fig. S2a:</u> Effect of renal ischemia/reperfusion in vehicle- and STZ-treated mice on desmin staining. Immunofluorescence images were captured using a Zeiss LSM 510 confocal microscope equipped with a 40x objective and laser excitation at 488 or 543 nm. Sham: sham-operated; IR: ischemia/reperfusion; STZ: streptozotocin; STZ/IR: streptozotocin/ischemia/reperfusion. Bars = 100 µm.

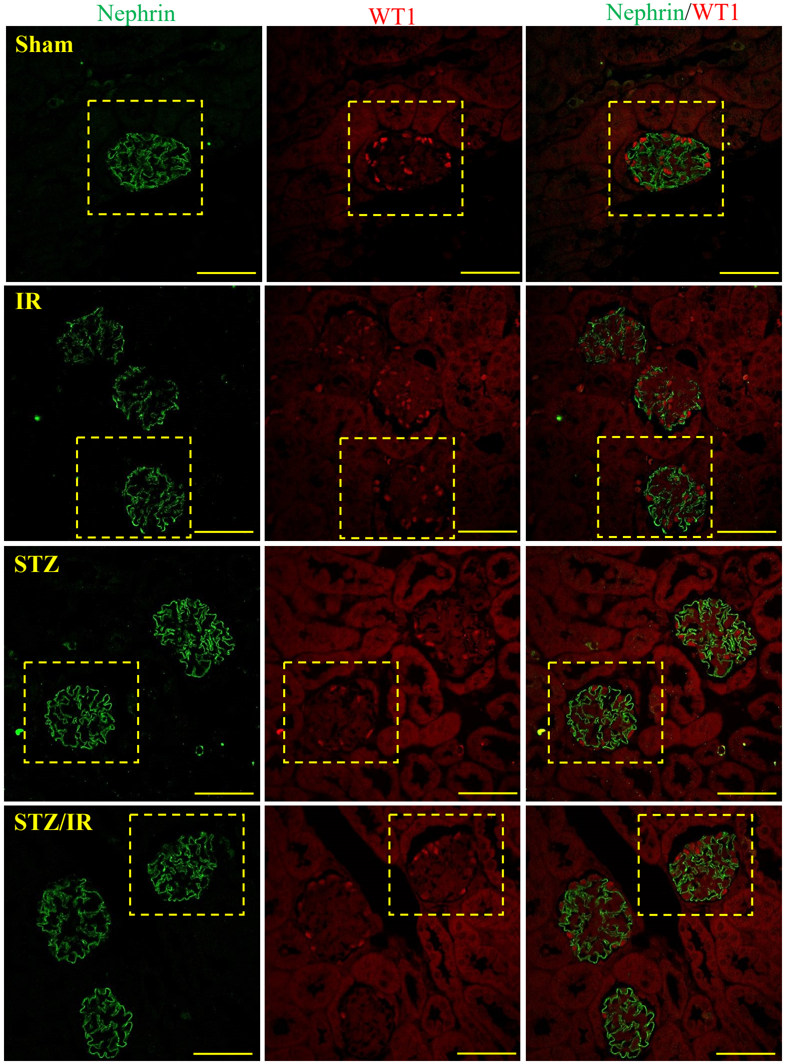

Supplementary Fig. S2b: Effect of renal ischemia/reperfusion in vehicle- and STZ-treated mice on nephrin and WT1 staining. Immunofluorescence images were captured using a Zeiss LSM 510 confocal microscope equipped with a 40x objective and laser excitation at 488 or 543 nm. Sham: sham-operated; IR: ischemia/reperfusion; STZ: streptozotocin; STZ/IR: streptozotocin/ischemia/reperfusion. Bars = 100 µm.

H&E staining

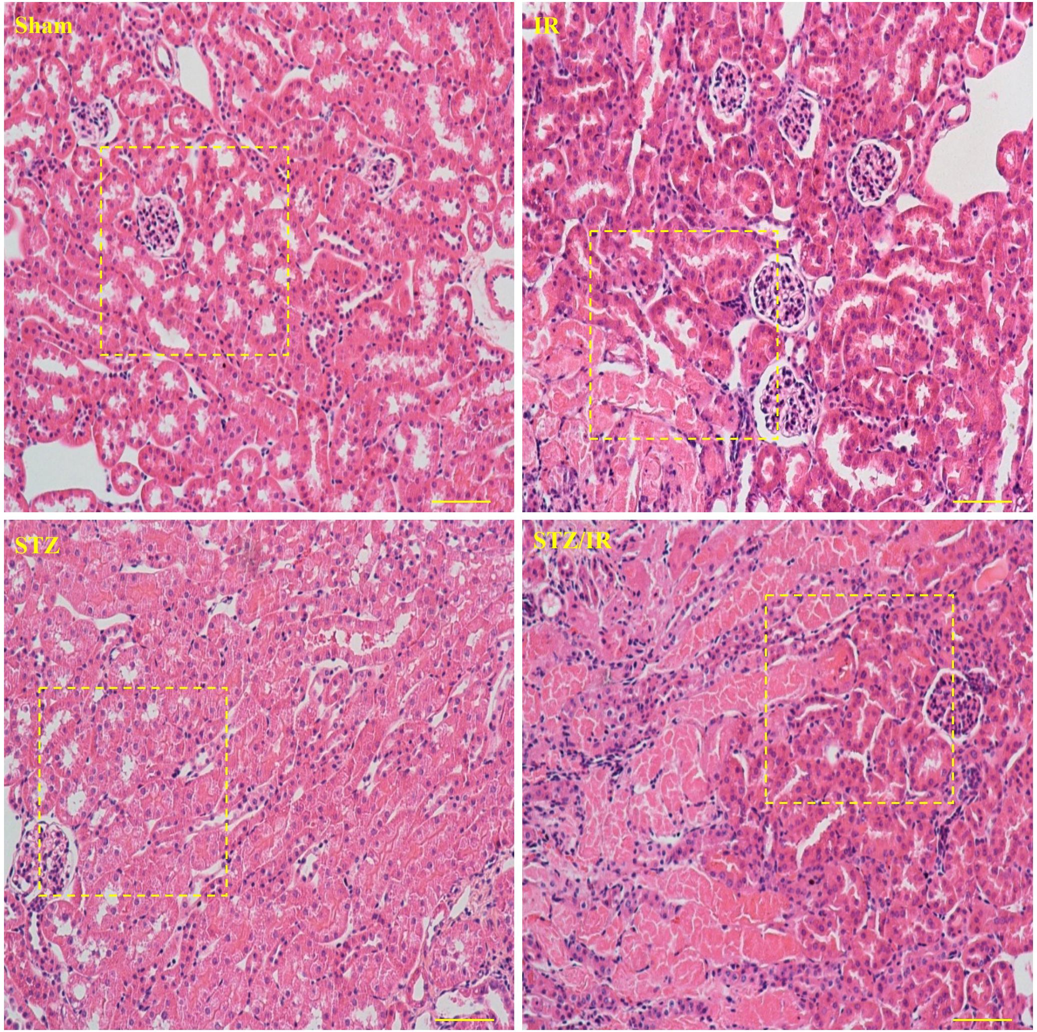

Supplementary Fig. S3a: Effect of renal ischemia/reperfusion in vehicle- and STZ-treated mice on tubular injury staining. Images were captured and analyzed using NIS-Elements (Nikon) software coupled to a light microscope equipped with a 20x objective (Eclipse 80i, Nikon, Tokyo, Japan). Sham: sham-operated; IR: ischemia/reperfusion; STZ: streptozotocin; STZ/IR: streptozotocin/ischemia/reperfusion. Bars = 100 µm.

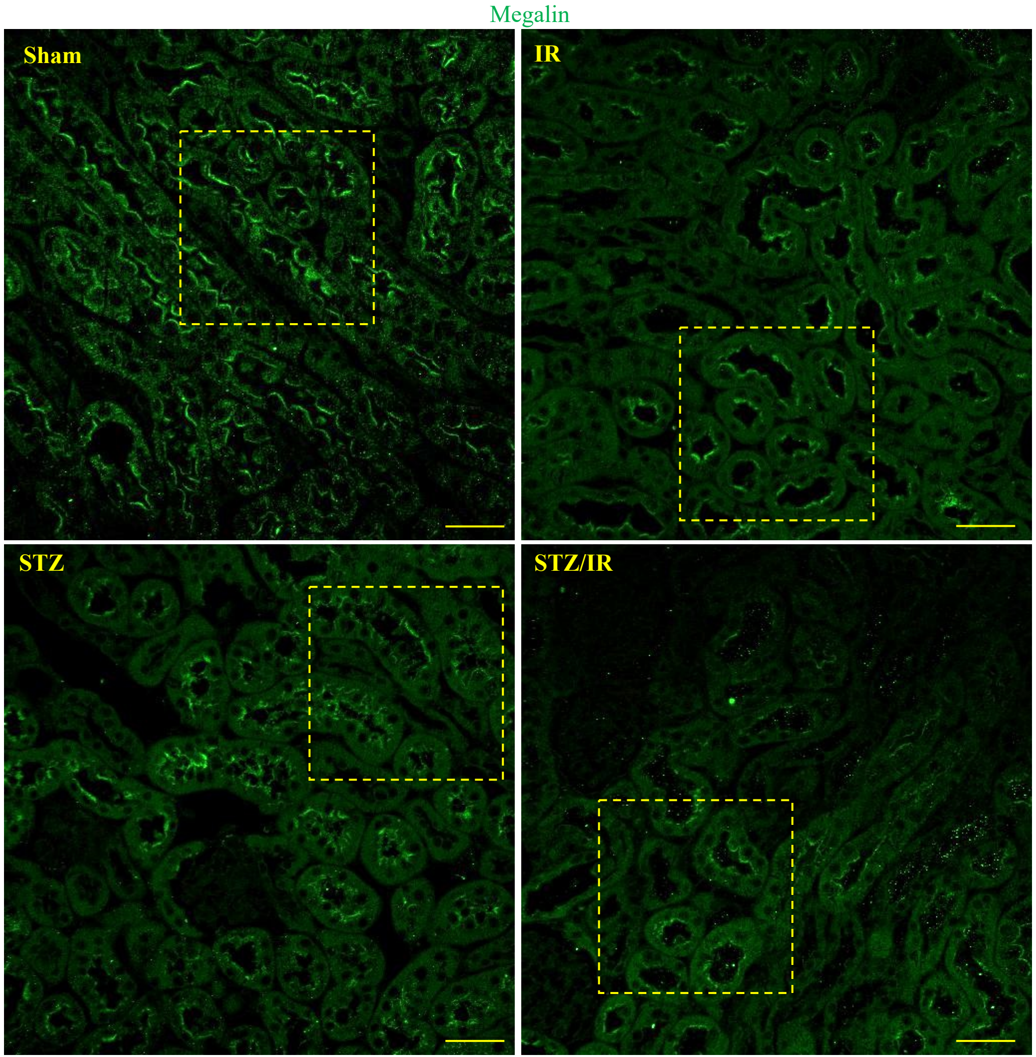

<u>Supplementary Fig. S3b</u>: Effect of renal ischemia/reperfusion in vehicle- and STZ-treated mice on megalin staining. Immunofluorescence images were captured using a Zeiss LSM 510 confocal microscope equipped with a 20x objective and laser excitation at 488 or 543 nm. Sham: sham-operated; IR: ischemia/reperfusion; STZ: streptozotocin; STZ/IR: streptozotocin/ischemia/reperfusion. Bars = 100 µm.

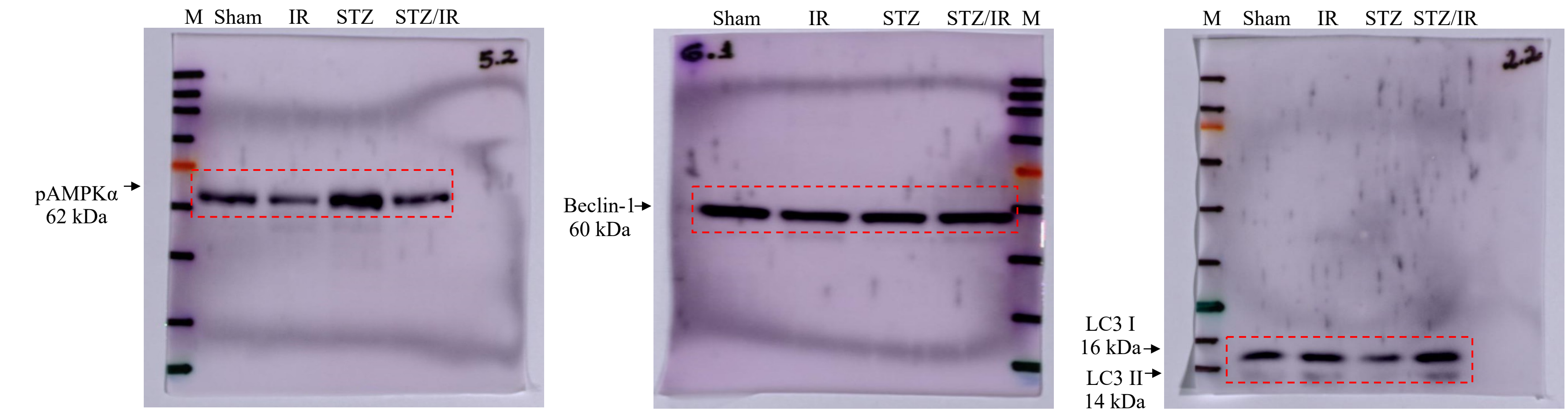

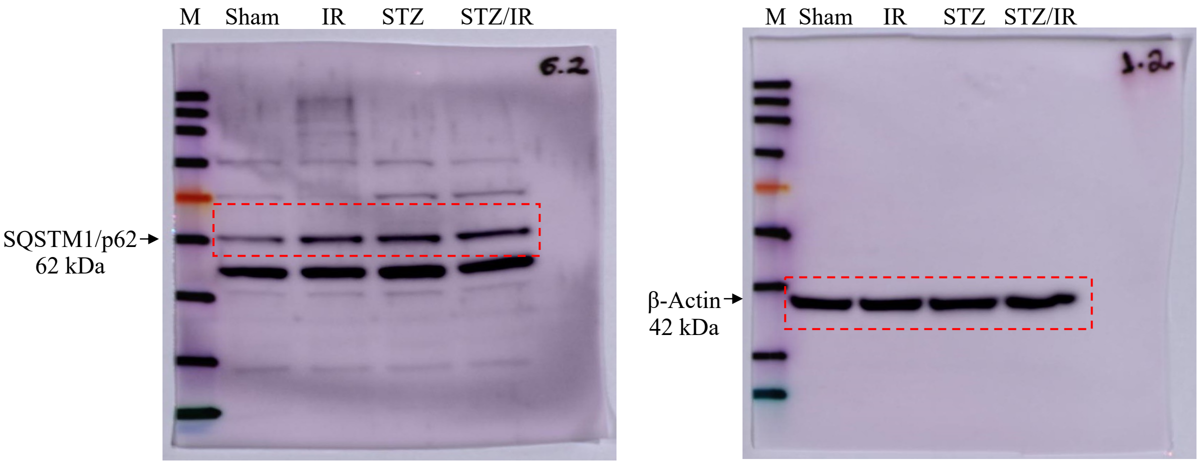

Supplementary Fig. S4: Effect of renal ischemia/reperfusion in vehicle- and STZ-treated mice on pAMPKα, Beclin -1, LC3 I and II, SQSTM1/p62 and β–actin protein expression. Representative full-length gel for each protein is presented. Sham: sham-operated; IR: ischemia reperfusion; STZ: streptozotocin; STZ/IR: streptozotocin/ischemia/reperfusion; M: marker.

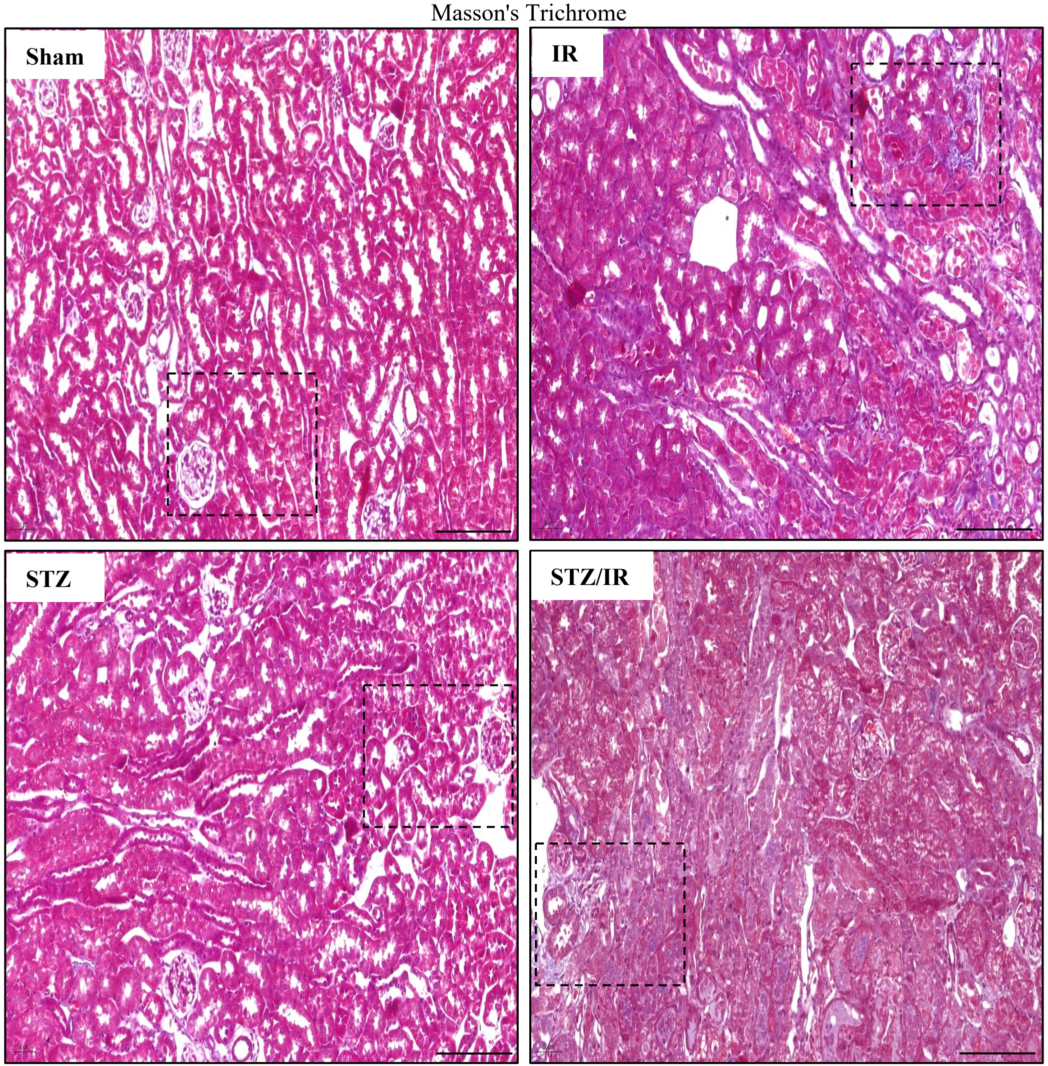

<u>Supplementary Fig. S5</u>: Effect of renal ischemia/reperfusion in vehicle- and STZ-treated mice on interstitial collagen volume. Images were captured and analyzed using NIS-Elements (Nikon) software coupled to a light microscope equipped with a 20x objective (Eclipse 80i, Sham: sham-operated; IR: ischemia reperfusion; STZ: streptozotocin; STZ/IR: streptozotocin/ischemia/reperfusion. Bars = 100 µm.
